# Regulation of *Escherichia coli* Group 2 Capsule Gene Expression: A Mini Review and Update

**DOI:** 10.3389/fmicb.2022.858767

**Published:** 2022-03-10

**Authors:** Esraa Aldawood, Ian S. Roberts

**Affiliations:** ^1^School of Biological Sciences, Faculty of Biology Medicine and Health, Manchester Academic Health Science Centre, University of Manchester, Manchester, United Kingdom; ^2^Clinical Laboratory Science, Collage of Applied Medical Science, King Saud University, Riyadh, Saudi Arabia

**Keywords:** K1 capsule, UTI, PR1 promoter, IHF, H-NS, BipA, SlyA

## Abstract

The expression of a group 2 capsule (K antigen), such as the K1 or K5 antigen, is a key virulence factor of *Escherichia coli* responsible for extra-intestinal infections. Capsule expression confers resistance to innate host defenses and plays a critical role in invasive disease. Capsule expression is temperature-dependent being expressed at 37°C but not at 20°C when outside the host. Group 2 capsule gene expression involves two convergent promoters PR1 and PR3, the regulation of which is critical to capsule expression. Temperature-dependent expression is controlled at transcriptional level directly by the binding of H-NS to PR1 and PR3 and indirectly through BipA with additional input from IHF and SlyA. More recently, other regulatory proteins, FNR, Fur, IHF, MprA, and LrhA, have been implicated in regulating capsule gene expression in response to other environmental stimuli and there is merging data for the growth phase-dependent regulation of the PR1 and PR3 promoters. The aim of the present Mini Review is to provide a unified update on the latest data on how the expression of group 2 capsules is regulated in response to a number of stimuli and the growth phase something that has not to date been addressed.

## Introduction

The expression of the capsule (K antigen) is a common feature of pathogenic *Escherichia coli* (Taylor and Roberts, 2005). There are over 80 different K antigens in *E. coli* which are classified based on biochemical and genetic properties into four groups, 1–4 (Whitfield and Roberts, 1999). The genetics, biosynthesis, and assembly of all four groups have been reviewed extensively with the regulation of expression of the K1 capsule used as a model to study group 2 capsule expression (Whitfield and Roberts, 1999; Whitfield, 2006; Corbett and Roberts, 2008).

The role of K1 capsule in urinary tract infections (UTI) has been studied both *in vitro* using the human bladder epithelial cell line PD07i and *in vivo* in murine model of UTI (Berry et al., 2009; Anderson et al., 2010; King et al., 2015). In the mouse model, the K1 capsule was found to be essential for the formation of intracellular bacterial communities (IBCs; Anderson et al., 2010), a key stage in the pathogenesis of UTI (Justice et al., 2004). Following growth in urine, strain UTI89 exhibited phase variable K1 capsule expression (Anderson et al., 2010; King et al., 2015) with the un-encapsulated bacteria being the initial colonizers of the bladder cells (King et al., 2015). Following internalization inside the PD07i bladder cells, the bacteria upregulated their capsule expression becoming encapsulated 2 h post-invasion (King et al., 2015). These data indicate that the stochastic regulation of capsule expression in urine may generate an un-encapsulated subpopulation that are the initial colonizers and pioneers of infection. Following escape into the cytosol capsule expression is switched on and IBCs are formed. As such, the regulation of K1 expression would appear critical in different stages of the UTI. The following sections summarize the genetic organization and what is known about regulation of Group 2 and examines the challenging unanswered questions.

## Genetic Organization of Group 2 Capsule Gene Clusters

**Figure 1:**
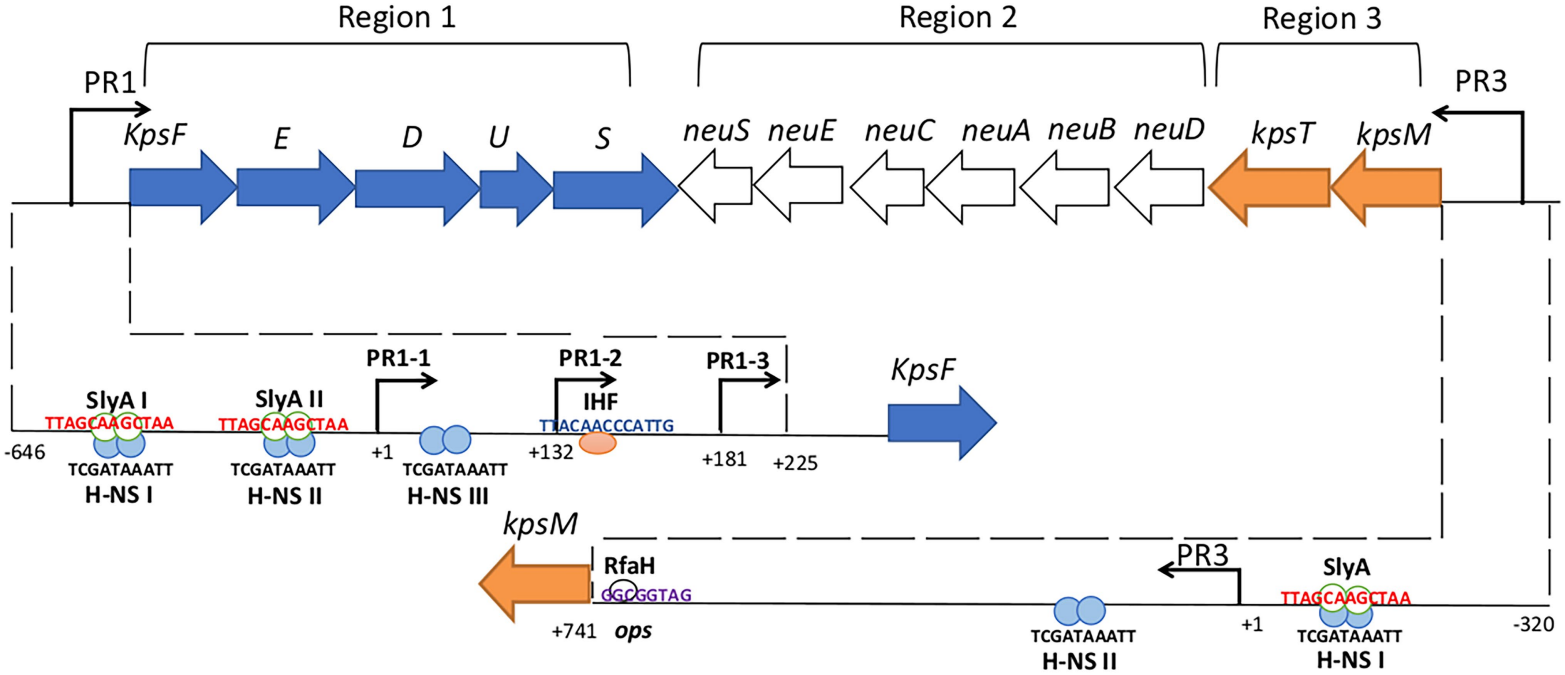
Genetic organization of *Escherichia coli* group 2 (K1) *kps* gene cluster. Group 2 *kps* clusters have two conserved regions 1 and 3 which are flank the serotype-specific region 2. Region 1 and 3 promoters (PR1 and PR3) are denoted by bent arrows. PR1 drives the transcription to region 1 genes while PR3 drives the transcription to region 3 genes and reads through to region 2 genes by RfaH antiterminator (white circle). The lower half of the figure dissects the PR1 and PR3 promoters. PR1 promoter contains three tandem promoters (PR1-1, PR1-2, and PR1-3) and the IHF (pink circle) binds to its consensus sequence at +140 while H-NS (blue circles) binds to three consensus sequences H-NS I, II, and III from position −224 to −134, −121 to −79, and +1 to +32. SlyA (green border white circles) and H-NS both bind to the H-NS I and II sites upstream of PR1-1 and the binding is not mutually exclusive. H-NS protects PR3 regions from −135 to −21 and +141 to +219 relative to transcription start site and SlyA overlaps H-NS upstream of PR1-3. Numbering indicates the position of the nucleotide relative to the transcription start site (modified from Aldawood, 2019).

Group 2 capsule gene clusters are composed of three regions (Figure 1). Region 1 and region 3, are conserved among all group 2 capsule gene clusters and encode proteins for polysaccharide export. Region 2, is serotype-specific and encodes proteins for the synthesis of each particular polysaccharide and its precursors (Roberts et al., 1988; Corbett and Roberts, 2008).

Region 1 contains six genes (*kpsFEDUCS*) organized in a single transcript that generates 8.0-kb polycistronic mRNA, which is processed to yield 1.3-kb *kpsS* specific transcript by an unknown mechanism (Figure 1; Roberts, 2002). The promoter region PR1 is located 225 bp upstream of *kpsF* (Cieslewicz and Vimr, 1996; Simpson et al., 1996) and contains three functional tandem promoters called PR1-1 (at +1), PR1-2 (at +132), and PR1-3 (at +181) that contribute to region 1 expression (Figure 1; Jia et al., 2017). Transcription from PR1-2 was found to be dependent on PR1-1 possibly through transcription-coupled DNA supercoiling, while PR1-3 was found to be an independent promoter (Jia et al., 2017). Integration Host Factor (IHF), a global regulator in *E. coli* that binds and bends DNA with a regulon of over 150 genes (Prieto et al., 2012), has a single binding site at +140 (Figure 1; Rowe et al., 2000).

Region 3 contains two genes (*kpsMT*) organized in a single transcriptional unit (Figure 1) encoding for the KpsMT ATP Binding Cassette transporter for the export of polysaccharide across the cytoplasmic membrane (Smith et al., 1990; Bliss and Silver, 1996). The PR3 promoter is 741 bp upstream to the initial codon of *kpsM* and has a typical *E. coli* σ70-10 consensus sequence but no −35 region (Stevens et al., 1997). An operon polarity suppressor (*ops*) sequence located 28 bp upstream of *kpsM* (Figure 1) facilitates RfaH-mediated read through transcription from PR3 that is essential for region 2 expression (Stevens et al., 1997; Whitfield and Roberts, 1999; Corbett and Roberts, 2008; Xue et al., 2009).

## Temperature Regulation of Group 2 Capsule Gene Clusters-the Roles of BipA, H-NS, SlyA, and IHF

Expression of group 2 capsule genes is temperature-regulated, with expression at 37°C in the host but not at 20°C when outside the host (Simpson et al., 1996; Rowe et al., 2000). This regulation is predominantly controlled at the level of transcription with coordinate regulation of the PR1 and PR3 promoter regions (Simpson et al., 1996; Rowe et al., 2000; Corbett et al., 2007). Both BipA and H-NS function in the temperature regulation of group 2 capsule gene expression (Rowe et al., 2000). BipA is a member of the ribosome-binding GTPase superfamily (Ero et al., 2016) important in ribosome assembly (Choi and Hwang, 2018). BipA regulates the transcription from PR1 and PR3 promoters at 37°C and 20°C, being required for maximal transcription from PR1 and PR3 at 37°C, but acting as a repressor at 20°C (Rowe et al., 2000). No BipA binding to either PR1 or PR3 was detectable indicating an indirect role for BipA. Recently, it has been proposed that at low temperature BipA senses changes in membrane fluidity and moderates LPS core biosynthesis gene expression to take on board alterations in fatty acid content and maintain membrane function (Choi et al., 2020). In which case the effects of a *bipA* mutation on group 2 capsule gene expression may be as a consequence of changes in LPS core biosynthesis that feedback *via* an as yet unknown system to regulate group 2 capsule gene expression and inappropriately switch on transcription at 20°C. This hypothesis linking LPS core gene expression to group 2 capsule gene expression is tempting when one considers that a *waaR* mutant defective in lipopolysaccharide outer core biosynthesis affects cell surface retention of group 2 capsules (Taylor et al., 2006).

**Figure 2:**
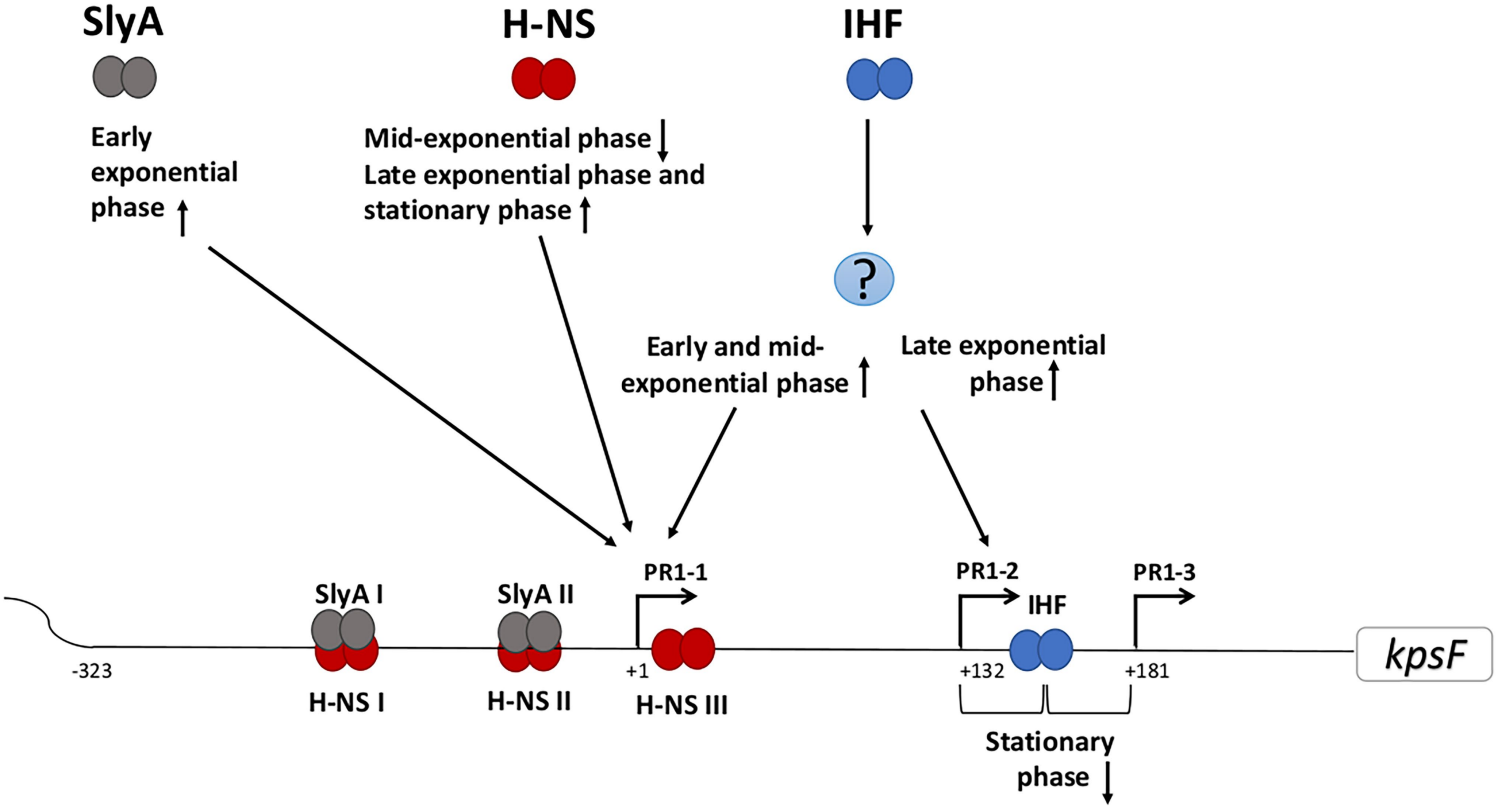
Schematic representation of the predicted regulation of the PR1 region of *E. coli* K1 by growth phase. Various regulatory proteins bind to the PR1 region that could activate (arrow pointing up) or repress (arrow pointing down) the transcription from the corresponding promoter at certain growth phase. The question mark denotes unknown regulator/regulators. IHF acts both directly and indirectly in regulating transcription from PR1. Indirectly, through as yet unknown protein(s), it activates both PR1-1 at early and mid-exponential phase and PR1-2 at the late exponential phase. In contrast by directly at +140, it represses PR1-2 and PR1-3 at stationary phase. H-NS I, II, and III denote the H-NS protected regions spanning through PR1 promoter from position −224 to −134, −121 to −79, and +1 to +32. SlyA overlaps H-NS upstream of PR1-1 but the binding is not mutually exclusive.

The global regulator H-NS regulates the temperature expression of a number of genes in *E. coli* typically acting to repress transcription at low temperature (Becker et al., 2007; Singh et al., 2014). In keeping with this H-NS represses transcription from both PR1 and PR3 at 20°C (Rowe et al., 2000) binding to both promoter regions with large DNase I footprints (Corbett et al., 2007; Xue et al., 2009). In PR1, three H-NS binding sites (I-III) were identified (Figure 2), while at PR3 two H-NS binding sites were identified either side of the transcriptional start site (Xue et al., 2009). Curiously at 37°C, H-NS was required for maximal transcription from both PR1 and PR3 with a *hns* mutant expressing reduced capsule expression at 37°C (Rowe et al., 2000). It has recently been shown that H-NS specifically represses PR1-1 at 20°C such that by switching off the major promoter in the PR1 region H-NS effectively silences transcription (Jia, 2014). In PR3, H-NS binding to the 3′ site was essential for preventing transcription at 20°C (Xue et al., 2009) with the long UTR modulating the extent of transcription that reached region 3 at 37°C (Xue et al., 2009).

SlyA regulates more than 30 genes in EIEC and Salmonella acting either as an activator or repressor (McVicker et al., 2011), controlling transcription of virulence genes by competition for binding sites with other proteins (Ellison and Miller, 2006; Ballesteros et al., 2019; Tian et al., 2020) often acting to counteract H-NS mediated repression (Banda et al., 2019). In the case of group 2 capsule gene clusters, SlyA was found to interact with H-NS to stimulate the transcription from PR1 and PR3 rather than merely acting to displace bound H-NS (Corbett et al., 2007; Xue et al., 2009). DNase I foot printing analysis of PR1 and PR3 promoters in the presence of both H-NS and SlyA revealed a DNase I footprint that was different to that generated by either SlyA or H-NS alone indicating that a reconfigured nucleoprotein complex is generated at the PR1 and PR3 promoters (Corbett et al., 2007; Xue et al., 2009). The observation that at 37°C, *hns* mutants have reduced capsule expression is in keeping with the requirement for H-NS for SlyA-mediated activation at 37°C (Corbett and Roberts, 2008). Likewise, the reduced SlyA expression at 20°C would be consistent with H-NS mediated repression at low temperatures (Corbett et al., 2007). Recent work has questioned the interplay between H-NS and SlyA at the PR1 promoter, suggesting that H-NS inhibits transcription at both 37°C and 20°C with SlyA functioning as an anti-repressor at 37°C (Jia, 2014). A more recent study (Aldawood, 2019) has suggested that H-NS and SlyA bind PR1 region at different stages during the growth phase (see below).

IHF is a global regulator in *E. coli* binding and bending DNA with a regulon of over 150 genes (Prieto et al., 2012). IHF was required for maximum transcription from PR1 at 37°C acting indirectly *via* an as yet unidentified regulator (Rowe et al., 2000; Jia et al., 2017). More recently the role of IHF in regulating transcription from PR1 has been expanded, in which IHF plays an additional direct role in the growth phase regulation of PR1 transcription (see below). No role for IHF has been established at PR3 (Stevens et al., 1997; Rowe et al., 2000).

## Growth Phase Regulation of Group 2 Capsule Gene Clusters-Interactions Between H-NS, SlyA, and IHF

Recent studies have demonstrated the growth phase-dependent expression of group 2 capsules and the differing roles of the three promoters in the PR1 region (Aldawood, 2019). The interaction between H-NS and SlyA at the PR1 promoters is growth phase-dependent with SlyA specifically activating transcription from PR1-1 in early exponential phase. The effect of H-NS was also growth phase-dependent having no effect on PR1-1 in early exponential phase, acting as a repressor at the mid-exponential phase before activating transcription as the cells enter stationary phase (Aldawood, 2019). While the concentration of SlyA varies during growth (Corbett et al., 2007), the level of H-NS does not but H-NS binding is sensitive to ionic strength and DNA superhelicity which change as the cells enter the stationary phase (Travers and Muskhelishvili, 2005; Dorman, 2006). As such the relative levels of SlyA and activity of H-NS during growth phase will impact on transcription from the PR1 region.

Both direct and indirect IHF regulation of individual promoters within the PR1 promoter region is growth phase-dependent (Aldawood, 2019). The indirect activation by IHF of PR 1-1 was only detected in the early and mid-exponential phase while at late exponential phase, IHF activates PR1-2 (Aldawood, 2019). In addition, the direct binding of IHF to its consensus binding site centered at +140 represses transcription from PR1-2 and PR1-3 upon entry to the stationary phase (Figure 2; Jia et al., 2017). It is known that the levels of IHF are growth phase-dependent being highest as the cells enter stationary phase (Martínez-Antonio et al., 2012; Lee et al., 2015) and have been shown that IHF is important to coordinate the expression of some virulence genes while adjusting to physiological changes associated with stationary phase transition (Mangan et al., 2006). The recent discovery that group 2 capsule expression is growth phase-dependent raises questions on how the growth phase may affect capsule expression during different stages of a UTI.

## FNR and Fur and Regulation of Capsule Gene Expression by Other Environmental Stimuli

The increased K1 expression in an avian pathogenic *E. coli* strain during growth in the presence of host serum and low oxygen has identified other environmental cues for capsule regulation (Ma et al., 2018). This regulation was attributed to two regulators, Fumarate Nitrate reductase Regulator protein (FNR) and Ferric Uptake Regulator (Fur; Ma et al., 2018). FNR is an oxygen sensor that allows facultative anaerobes to adjust to O_2_ deprivation (Green et al., 2009), while Fur is an iron sensing regulator controlling expression of more than 80% of the serum-upregulated genes in *E. coli* (Huja et al., 2014). FNR was shown to bind to PR1 and PR3 promoters to activate the transcription under low oxygen serum conditions, while Fur represses transcription from both PR1 and PR3 promoters in an iron-replete medium, with growth in iron-depleted serum enhancing the expression of capsule genes by relieving the Fur repression (Ma et al., 2018). Fur was shown to bind to an overlapping site with FNR at PR3 promoter region, no Fur binding has been detected at PR1 suggesting that the role of Fur repression on PR1 could be indirect (Ma et al., 2018). No study has yet investigated which promoter in the PR1 region is affected by Fur and FNR.

## Roles for Additional Regulators of Capsule Gene Expression-MprA and LrhA

Both MprA and LrhA were found to affect transcription from both PR1 and PR3 promoters (Goh et al., 2017). MprA activates transcription from both PR1 and PR3 promoters probably indirectly as studies were unable to show direct binding of MprA to either PR1 or PR3 promoters (Arshad et al., 2016; Goh et al., 2017). The overexpression of LrhA reduced the transcription of capsule biosynthesis genes indicting that LrhA represses the transcription from both PR1 and PR3 promoters. It is unknown if LrhA binds directly to repress the transcription from PR1 and PR3 promoters and which promoter in the PR1 region is specifically affected by MprA and LrhA. Currently, it is unknown how these two regulators interact with other well-characterized regulators and how their activity is affected by environmental stimuli.

## Discussion

The regulation of group 2 capsule genes expression is complex including multiple promoters controlled by various proteins that may respond to different triggers. Temperature regulation is best understood and on one level is straight forward, temperature-dependent transcriptional regulation. The complexity and nuances of the system lie in the number of regulatory proteins involved and their relative interactions. At 20°C, the capsule is switched off by H-NS binding to PR1 and PR3 promoters. This silencing of transcription could be by the initial binding and nucleation at the binding site or due to H-NS bridging (Lang et al., 2007; Xue et al., 2009). The role of BipA in this transcriptional repression at 20°C is almost certainly indirect and we would propose that this may be *via* a similar mechanism by which BipA senses changes in membrane fluidity at low temperature and moderates LPS core gene expression (Choi et al., 2020). The activation of transcription from PR1 and PR3 regions is more complex. The data about the interactions between SlyA and H-NS at 37°C are conflicting, and it is still unresolved as to whether SlyA interacts with H-NS to stimulate transcription or merely acts to overcome H-NS repression. From the recent detailed analysis of the PR1 region, it is now clear that expression from this promoter region is growth phase-dependent with H-NS and SlyA acting on PR1-1 at different growth stages suggesting that SlyA and H-NS do not function simultaneously when regulating the transcription from PR1-1 (Aldawood, 2019).

H-NS concentration does not change during growth (Travers and Muskhelishvili, 2005; Dorman, 2006). Therefore, the growth phase-dependent effect of H-NS could be attributed to the global DNA supercoils that change during the growth since it is known H-NS binding can be affected by the DNA topology (Dorman, 2004, 2006; Lim et al., 2014). One can speculate that in the mid-exponential phase, both H-NSII and H-NSIII would be occupied by H-NS allowing bridge formation, DNA looping, and transcription inhibition of PR1-1 (Figure 2; Liu et al., 2010). The mechanism by which H-NS activates PR1-1 in the stationary phase is unknown but it was suggested that H-NS may act as an architectural component facilitating the recognition of PR1-1 functional elements by RNA polymerase (Aldawood, 2019). IHF appears to play a critical role in regulating the growth phase-dependent transcription from each promoter in the PR1 (Jia et al., 2017; Aldawood, 2019). The proposed repression model of PR1-3 is by DNA wrapping at +140, which overlaps the −35 of PR1-3 when the IHF concentration increases inside the cell before the entry to stationary phase (Jia et al., 2017). Moreover, the binding of IHF at this position may create a roadblock to block transcription initiating from PR1-2 (Jia et al., 2017). The interesting finding, that the same regulatory protein (H-NS, SlyA, and IHF) variably affects each promoter in the PR1 region according to the growth phase of the cell, allows speculation that within the host, the regulation is also variable according to the niches the bacterium may encounter. Currently, it is unknown whether expression from PR3 is also growth phase regulated, but our hypothesis is that this is likely since coordinate regulation of PR1 and 3 would be necessary to ensure cell surface capsule expression and avoid the wasteful synthesis of capsule biosynthesis proteins and polysaccharide inside the cell. Examining the role of each regulatory protein in regulating PR1 promoters in the diverse niches within the host starting from the gut to the urethra then growing in the urine environment followed by invading the bladder cells resulting in IBC formation is a fascinating area that has never been studied.

The regulation of the transcription of PR1 is expected to be more complicated as two additional transcriptional regulators (MprA and LrhA) have been found to affect transcription from PR1 (Goh et al., 2017). While it is more likely that MprA regulates capsule expression indirectly, no investigations have been carried out to find if LrhA binds directly to the PR1 region. Additionally, we hypothesize that the increased capsule expression in the presence of low oxygen and serum in avian pathogenic *E. coli* strains is likely to be true for urinary pathogenic *E. coli*, with similar roles for FNR and Fur. However, at this stage, it is unknown where FNR binds relative to PR1 promoters. We predict that stochastic expression of the *E. coli* K1 capsule (King et al., 2015) is a consequence of interaction of multiple regulatory proteins acting at the PR1 promoter region combined with growth phase-dependent effects. As such, small changes in the relative level of individual regulatory proteins coupled to the nutrient status of the individual cell could affect capsule expression. These phenomena have been observed with other bistable expression systems (Kærn et al., 2005; Dubnau and Losick, 2006; Robert et al., 2010).

In conclusion, the regulation of expression of group 2 capsules involves multiple regulatory proteins acting on both PR1 and PR3. The future challenges are to further refine and dissect the complex regulatory circuitry of group 2 capsule expression in *E. coli*. To begin to translate these *in vitro* derived data into our understanding of capsule expression during both carriage and infection in the host and to appreciate how growth rate *in vivo* could affect the spatial and temporal expression of capsules and interactions with host cells.

## Author Contributions

All authors listed have made a substantial, direct, and intellectual contribution to the work and approved it for publication.

## Funding

EA gratefully acknowledges funding for a PhD scholarship from the King Saud University, Riyadh, Saudi Arabia.

## Conflict of Interest

The authors declare that the research was conducted in the absence of any commercial or financial relationships that could be construed as a potential conflict of interest.

## Publisher’s Note

All claims expressed in this article are solely those of the authors and do not necessarily represent those of their affiliated organizations, or those of the publisher, the editors and the reviewers. Any product that may be evaluated in this article, or claim that may be made by its manufacturer, is not guaranteed or endorsed by the publisher.
